# Gallstone Ileus Post-cholecystectomy: A Case Review

**DOI:** 10.7759/cureus.33345

**Published:** 2023-01-04

**Authors:** Nader A Helmy, Ondrej Ryska

**Affiliations:** 1 Department of General Surgery, Cairo University Teaching Hospitals, Kasr Al Aini, Cairo, EGY; 2 Department of General Surgery, Royal Lancaster Infirmary, University Hospitals of Morecambe NHS Trust, Lancaster, GBR

**Keywords:** small bowel obstruction, rigler’s triad, post-cholecystectomy, biliary-enteric fistula, gallstone ileus

## Abstract

Gallstone ileus is an unusual cause of small bowel obstruction, in general, let alone after cholecystectomy. It occurs in patients with chronic calculous cholecystitis and gallstones who develop a cholecystoduodenal fistula over time. The diagnosis is made based on clinical presentation and examination findings and is confirmed with the use of radiological modalities, such as computed tomography (CT) scan, which has been proven to be the most sensitive investigation in diagnosis. Here, we present a case of gallstone ileus that occurred 25 years after laparoscopic cholecystectomy. CT scan on admission showed adhesional small bowel obstruction given the patient’s previous abdominal surgery. The patient was managed conservatively as per guidelines for the management of adhesional small bowel obstruction for 72 hours. Obstructive symptoms did not resolve despite all conservative measures, and a gastrografin challenge showed no contrast reaching the colon. Hence, the patient underwent an exploratory laparotomy to manage his ongoing bowel obstruction. Laparotomy revealed gallstone ileus as the cause of obstruction. This case highlights the importance of considering gallstone ileus in the differential diagnosis for patients who present to the emergency department with small bowel obstruction even years after cholecystectomy. Post-cholecystectomy gallstone ileus is very rare with very few cases reported in the literature. This condition poses diagnostic challenges both because of its rarity and because the gallbladder had been previously removed. A high index of suspicion by the surgeon is needed for diagnosis.

## Introduction

Gallstone ileus is a relatively uncommon cause of small bowel obstruction. The underlying cause is longstanding chronic cholecystitis which leads to the formation of a biliary-enteric fistula, which eventually causes stone migration and bowel obstruction. It typically occurs in elderly females [1]. It is considered an uncommon complication caused by recurrent episodes of gallbladder inflammation, or, rarely, gallbladder cancer [2]. The mortality rate for gallstone ileus can be up to 18% in elderly patients, though this can be related to the associated comorbidities in this patient population and the delayed presentation [1]. Early diagnosis as well as suitable and timely surgical intervention are considered the two most important predictors of better prognosis [3]. The diagnosis of gallstone ileus is made based on the clinical presentation with the aid of radiographic investigations, with computed tomography (CT) scan with contrast being the most sensitive radiographic modality [4].

Rigler’s triad, a pathognomonic radiological triad, is not always picked up by plain X-ray or ultrasound but is more commonly seen on CT scans with higher accuracy and sensitivity [4]. Rigler’s triad consists of radiological signs of small bowel obstruction, pneumobilia (or air in the biliary tree), and ectopic calcified gallstone, usually identified in the right iliac fossa. Two out of these three radiological signs are sufficient for the diagnosis of gallstone ileus [4].

The treatment of this condition is the surgical removal of the gallstone causing the obstruction, via a proximal enterotomy, with or without excision of the underlying biliary-enteric fistula during the same procedure depending on the patient’s performance status and general condition [5]. According to the literature, enterolithotomy, without exploring the fistula, is the most commonly applied surgical technique in the management of gallstone ileus, followed by elective laparoscopic cholecystectomy after a few weeks. Enterolithotomy combined with cholecystectomy and fistulectomy in the same setting is only indicated in highly selected patients [6].

We present this case of gallstone ileus because it occurred 25 years post-laparoscopic cholecystectomy. The incidence of gallstone ileus as a cause of small bowel obstruction is low, as reviewed in the literature, let alone decades post-cholecystectomy. In our patient, we believe that the presence of small bowel diverticulosis (another uncommon condition that was diagnosed intraoperatively) helped keep his gallstone in the small bowel for years, which then dislodged to cause acute small bowel obstruction.

## Case presentation

Clinical presentation

An 81-year-old male patient presented to the A&E Department at Royal Lancaster Infirmary, University Hospitals of Morecambe Bay on April 13, 2021, with central abdominal pain for one week, which worsened in the last 24 hours prior to presentation. The pain was dull aching, located in the upper-central abdomen, and not radiating to any other point. He also complained of vomiting for four days. This was associated with anorexia and struggling to open his bowels. He described progressive constipation, which was interrupted with overflow diarrhea two days prior to presentation, followed by absolute constipation for the following 48 hours (till presentation).

History

The patient reported a medical history of hypertension, psoriasis, and Raynaud’s. In addition, he reported an abdominal surgical history of laparoscopic cholecystectomy 25 years prior to presentation in 1996. There was no other surgical history of relevance. The patient was a non-smoker and consumed two to three units of alcohol a week.

Clinical examination

On clinical examination, the patient’s chest was clear with bilateral equal air entry. His abdomen was noted to be soft, lax, moderately distended, and with mild periumbilical tenderness. There were no signs of generalized peritonitis and no cough on impulse on all hernial orifices.

Investigations

The patient’s blood tests revealed mild leukocytosis (white cell count 11 × 10^9^ cells/L), mildly elevated C-reactive protein (161 mg/L), and renal functions suggestive of prerenal acute kidney injury. The rest of the blood parameters were normal. Testing for coronavirus disease 2019 was negative. Abdominal X-ray revealed dilated small bowel loops, collapsed colon, and no clear transition zone.

**Figure 1:**
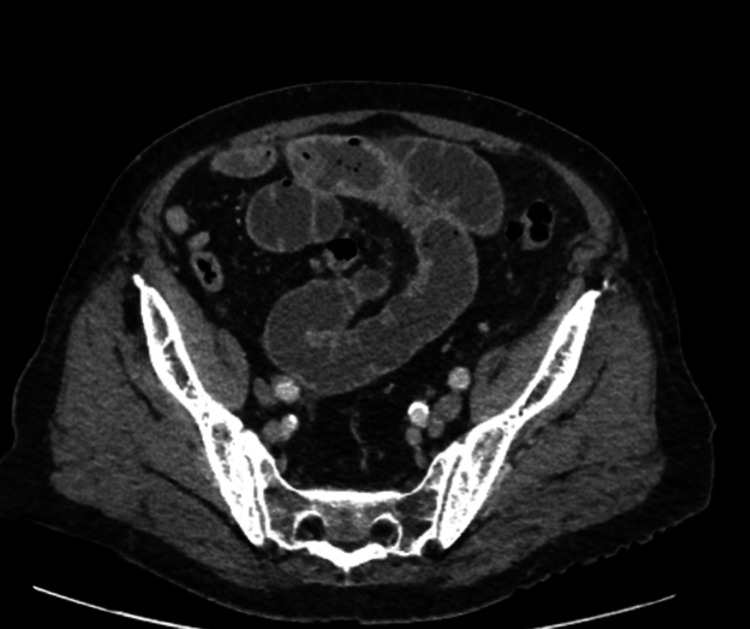
Computed tomography scan on admission showing the distended proximal small bowel and collapsed colon.

**Figure 2:**
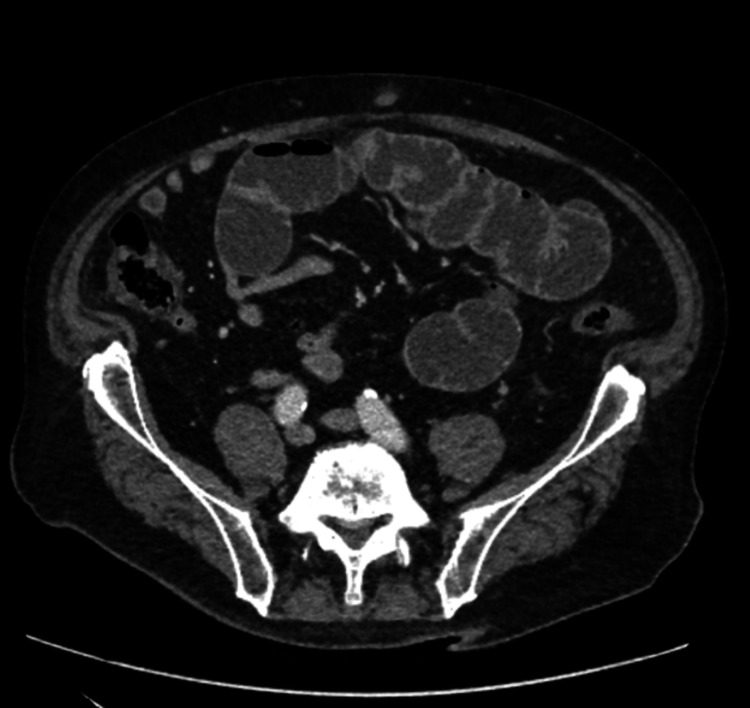
Computed tomography scan on admission showing the distended small bowel and transition zone in the retro-umbilical region.

He was admitted to the acute surgical unit for monitoring. Intravenous fluids and antibiotics were prescribed. An urgent abdominopelvic CT scan was requested. His renal functions improved with fluid resuscitation. On the CT scan, the stomach, duodenum, jejunum, and very proximal ileum were distended, with a clear transition zone between the distended and collapsed small bowel loops seen in the proximal ileum in the retro-umbilical region (Figures 1, 2). There was a slight thickening of the small bowel at the transition zone, but no definite lesion was demonstrated at this point. Beyond this transition point, both the small and large bowels were collapsed. No radiological features suggested internal herniation or volvulus. Hence, the small bowel obstruction was likely to be adhesional secondary to his previous abdominal surgery. The previous cholecystectomy was noted.

Management

**Figure 3:**
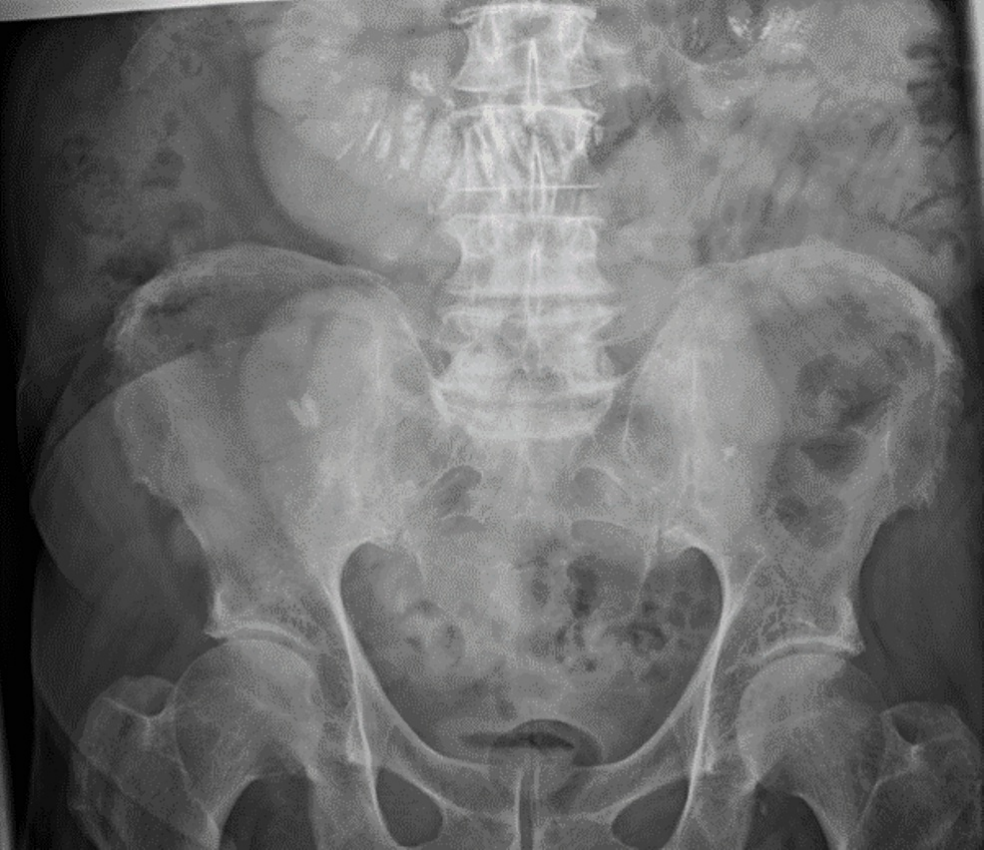
Gastrografin challenge with no contrast seen in the colon.

We kept our patient admitted and started conservative treatment with a nasogastric tube (NGT) and intravenous fluids as per guidelines in the management of adhesional small bowel obstruction. NGT output was significant (1,620 mL immediately upon insertion). The patient felt better, and the NGT was kept on free drainage. The NGT output persisted at high levels (~1,400 mL per 24 hours), and the patient did not show any signs of clinical improvement. His abdomen remained distended, and he did not open his bowels. Hence, we decided to perform a gastrografin challenge followed by an abdominal X-ray as per guidelines on April 15, 2021. Unfortunately, his abdominal X-ray (done at eight and 24 hours post-gastrografin ingestion) did not show any contrast reaching the colon, as shown in Figure 3, and there was no clinical improvement.

The decision was made to take the patient to the theater for exploratory laparotomy for unresolving adhesional bowel obstruction despite 72 hours of conservative treatment.

Operation

A periumbilical midline incision (mini-laparotomy) was made. Small bowel obstruction with a transition zone between the collapsed ileum and distended jejunum was noted. A 2-cm obstructing gallstone was seen at the transition zone (~2.5 m distal to duodenojejunal junction). Worth noting, significant small bowel diverticulosis was also noted intraoperatively (could be where the stone was impacted for years prior to dislodging to obstruct the small bowel lumen), as well as sigmoid diverticulosis, and healthy small bowel all through. No other obstructing pathology was detected.

A formal abdominal exploration revealed the above findings. Enterotomy was done just proximal to the site of obstruction. A 2-cm gallstone was retrieved through the enterotomy. The rest of the small bowel appeared healthy and viable. The enterotomy was then closed in a stricturoplasty-like pattern (Heineke-Miculikz-like) using 3/0 visiblack in two layers. Peritoneal lavage and suction were done prior to the insertion of rectus sheath catheters and closure of the abdominal wall in layers.

Postoperative management

The patient was allowed oral fluids and a soft diet on postoperative day one as per the Enhanced Recovery After Surgery (ERAS) program. He passed flatus on postoperative day one; however, because the NGT drained 1.2 L, it was not removed. On postoperative day two, the NGT was spigotted and frequently aspirated. On postoperative day three, urinary catheter, rectus sheath catheters, and NGT were removed, and a normal diet was allowed. The patient was discharged home on postoperative day four after opening his bowels and eating and drinking freely without any vomiting. His blood tests were back to normal after a full recovery.

## Discussion

Classically, patients presenting with gallstone ileus have a long-term history of ongoing chronic with recurrent episodes of acute cholecystitis, leading to adherence of the gallbladder to the duodenum which eventually causes a cholecystoduodenal fistula. Most patients (96.5%) presenting with gallstone ileus have an underlying cholecystoduodenal fistula [7].

Another indirect cause is a surgical technique known as subtotal cholecystectomy, which is practiced more and more recently. Subtotal cholecystectomy is an acceptable approach to managing gallbladder stones when there is severe inflammation of the gallbladder. It is a safe bailout option to avoid hazardous dissection of a plastered Calot’s triangle and to avoid iatrogenic injuries. However, when performing this procedure, it must be ensured that the stump of the gallbladder is free of any remnant stones as complications, such as choledocholithiasis, gallstone pancreatitis, acute cholecystitis, persistent biliary fistula, and even gallstone ileus, have been documented in relation to this procedure [8].

Similar to our patient, Zens et al. reported a rare case of a 91-year-old female who presented with small bowel obstruction, which turned out to be secondary to gallstone ileus 30 years after cholecystectomy. However, it is noteworthy that this patient had a large duodenal diverticulum, which could have extruded the gallstone that later caused the intestinal obstruction. This likelihood is also supported by the absence of pneumobilia in this case [9]. Similarly, our patient was found to have multiple small bowel diverticula intraoperatively, which could have extruded his gallstone that caused the small bowel obstruction decades after cholecystectomy.

Unlike the presentation of our patient, Inal et al. reported that the most common site of gallstone impaction is the terminal ileum [10,11].

Other potential, though less common, sites of impaction include the duodenum (Bouveret syndrome, leading to gastric outlet obstruction), stomach, jejunum, proximal ileum, or even the colon. Table 1 shows the incidence of the impaction of the gallstone in different locations according to Nuno-Guzman et al. [7,11].

**Table 1:** Sites of gastrointestinal obstruction in patients with gallstone ileus.

Site	Incidence
Duodenum	0–10.5%
Stomach	0–20%
Jejunum	0–50%
Jejunum/Proximal ileum	0–50%
Terminal ileum	0–89.5%
Colon	0–8.1%
Undetermined	0–25%

It is worth mentioning that laparoscopic and laparoscopic-assisted enterolithotomy may have a role in the management of gallstone ileus and may have advantages when compared to open surgery in certain patients. The laparoscopic approach essentially emulates the classic open enterolithotomy approach. The potential benefits of the laparoscopic approach include shorter hospital stay, less postoperative pain, and reduced morbidity and complications caused by laparotomy [11,12]. The prerequisites when attempting keyhole surgery in these patients include a competent experienced surgeon capable of intracorporeal knotting. There are many precautions that should be taken during laparoscopic surgery in these patients, such as the effect of CO_2_ insufflation and the risks associated with high insufflation pressures, especially in the elderly population, bowel edema, secondary to the ongoing bowel obstruction, difficult closure of the incision site, and bowel distension, which may actually preclude safe port entry. Intraoperatively, the entire small bowel must be thoroughly examined to exclude further stones, just as with the open technique [11,13].

Laparoscopic-assisted extracorporeal enterolithotomy may act as a bridge between both techniques, avoiding some of the risks associated with the total laparoscopic approach. It is also ideal if an expert surgeon with intracorporeal knotting is unavailable [11,14].

Moberg et al. retrospectively compared the results of laparoscopic-assisted and open enterolithotomy in a case series of 32 patients. They reported that patients who were managed laparoscopically suffered from relatively minor complications with similar operative times to those managed with open surgery. Worth mentioning, all patients in the laparoscopic group in this study had no previous abdominal surgical history [11,15].

On the other hand, only 10% of gallstone ileus cases were managed laparoscopically in the study by Halabi et al. [1]. Higher rates of conversion from laparoscopic to open were reported as well, especially when a one-stage procedure was attempted [1,11].

Therefore, the laparoscopic approach in the management of bowel obstruction caused by gallstone ileus may be considered in the management of carefully selected patients, when a well-trained, experienced laparoscopic surgeon is available, and may result in improved and expedited postoperative recovery [11].

## Conclusions

Although post-cholecystectomy gallstone ileus is a rare form of small bowel obstruction, all surgeons must be aware of its existence, because the diagnosis requires a very high index of suspicion, even decades post-cholecystectomy, which can be confirmed with an abdominal CT showing elements of Rigler’s triad. Familiarity with this entity and knowing its clinical presentation and radiological findings can lead to earlier diagnosis and better management.

The management of gallstone ileus is essentially surgical, and the choice of surgical procedure depends on the baseline general condition of the patient, the intraoperative findings, and the level of training of the main operating surgeon. Simple enterolithotomy and extraction of the gallstone is the treatment of choice for most cases and is the most beneficial for patients with poor overall health. Moreover, laparotomy should involve a systematic and meticulous search for the presence of further enteric stones.
